# COVID-19 and the Vasculature: Current Aspects and Long-Term Consequences

**DOI:** 10.3389/fcell.2022.824851

**Published:** 2022-02-15

**Authors:** Berenice Martínez-Salazar, Melle Holwerda, Chiara Stüdle, Indre Piragyte, Nadia Mercader, Britta Engelhardt, Robert Rieben, Yvonne Döring

**Affiliations:** ^1^ Division of Angiology, Swiss Cardiovascular Center, Inselspital, Bern University Hospital, University of Bern, Bern, Switzerland; ^2^ Department for BioMedical Research (DBMR), University of Bern, Bern, Switzerland; ^3^ Theodor Kocher Institute, University of Bern, Bern, Switzerland; ^4^ Institute of Anatomy, University of Bern, Bern, Switzerland; ^5^ Centro Nacional de Investigaciones Cardiovasculares (CNIC), Madrid, Spain; ^6^ Bern Center of Precision Medicine BCPM, University of Bern, Bern, Switzerland; ^7^ Institute for Cardiovascular Prevention (IPEK), Ludwig-Maximilians-University Munich (LMU), Munich, Germany; ^8^ DZHK (German Centre for Cardiovascular Research), Partner Site Munich Heart Alliance, Munich, Germany

**Keywords:** SARS-CoV-2, COVID-19, long COVID-19 syndrome, endothelium, heart

## Abstract

Severe Acute Respiratory Syndrome Coronavirus 2 (SARS-CoV-2) was first identified in December 2019 as a novel respiratory pathogen and is the causative agent of Corona Virus disease 2019 (COVID-19). Early on during this pandemic, it became apparent that SARS-CoV-2 was not only restricted to infecting the respiratory tract, but the virus was also found in other tissues, including the vasculature. Individuals with underlying pre-existing co-morbidities like diabetes and hypertension have been more prone to develop severe illness and fatal outcomes during COVID-19. In addition, critical clinical observations made in COVID-19 patients include hypercoagulation, cardiomyopathy, heart arrythmia, and endothelial dysfunction, which are indicative for an involvement of the vasculature in COVID-19 pathology. Hence, this review summarizes the impact of SARS-CoV-2 infection on the vasculature and details how the virus promotes (chronic) vascular inflammation. We provide a general overview of SARS-CoV-2, its entry determinant Angiotensin-Converting Enzyme II (ACE2) and the detection of the SARS-CoV-2 in extrapulmonary tissue. Further, we describe the relation between COVID-19 and cardiovascular diseases (CVD) and their impact on the heart and vasculature. Clinical findings on endothelial changes during COVID-19 are reviewed in detail and recent evidence from *in vitro* studies on the susceptibility of endothelial cells to SARS-CoV-2 infection is discussed. We conclude with current notions on the contribution of cardiovascular events to long term consequences of COVID-19, also known as “Long-COVID-syndrome”. Altogether, our review provides a detailed overview of the current perspectives of COVID-19 and its influence on the vasculature.

## 1 Introduction

Severe acute respiratory syndrome coronavirus 2 (SARS-CoV-2) belongs to the Betacoronavirus family and was identified in December 2019/January 2020 in lung tissue of patients with symptoms resembling severe pneumonia, including acute respiratory distress syndrome (ARDS). This new disease was named Corona Virus disease 2019 (COVID-19) (Zhu N. et al., 2020). SARS-CoV-2 shares 79% similarity with SARS-CoV, another Coronavirus responsible for the SARS outbreak in 2002–2004 (Drosten et al., 2003; Kuiken et al., 2003; Lu et al., 2020). The single-stranded positive-sense viral RNA genome of SARS-CoV-2 encodes around 29 proteins from which 4 are structural proteins: spike (S), envelope (E), membrane-associated (M), and nucleocapsid (N) protein (Yao H. et al., 2020). The S protein is most important for the virus to enter the cell via its interaction with Angiotensin Converting Enzyme II (ACE2) and the transmembrane serine protease 2 (TMPRSS2) that cleaves the viral S protein facilitating its entrance into the cell (Hoffmann et al., 2020). Both N and M proteins are responsible for binding and packing the viral genome (Lu et al., 2021). The N protein further induces a strong immune response fostering the production of specific antibodies (IgA, IgG, and IgM) which are also useful for diagnostic testing of COVID-19 (Zeng et al., 2020; Barlev-Gross et al., 2021; De Marco Verissimo et al., 2021).

**FIGURE 1:**
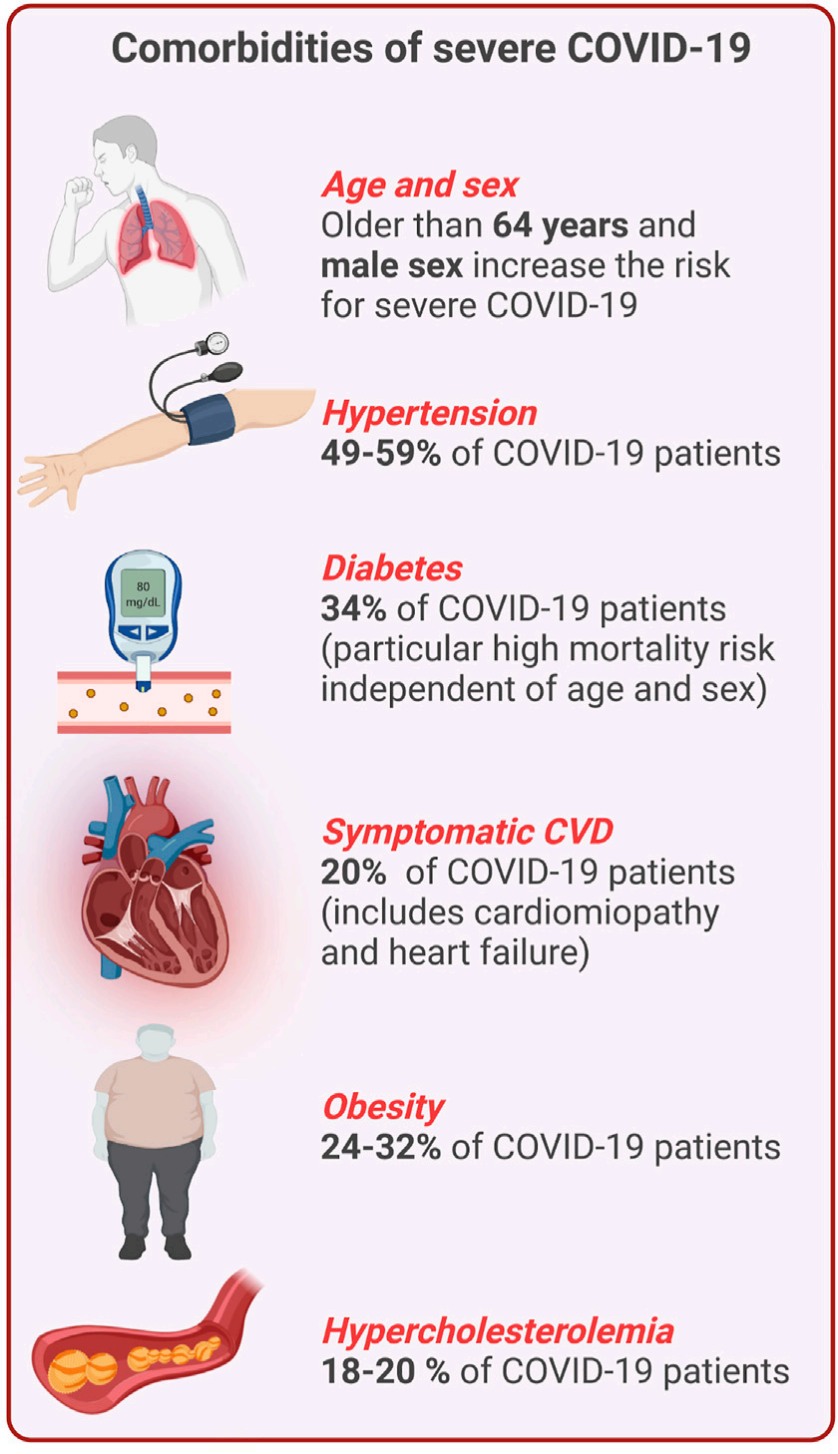
Overview of comorbidities of severe COVID-19 and CVD. Percentages indicate the frequency of certain comorbidities and COVID-19 (This figure was made with biorender.com).

Approximately half of the patients with COVID-19 are asymptomatic or mildly symptomatic, but 3–10% of patients with COVID-19 require hospitalization, of which up to 20% may suffer from severe disease leading to a high mortality rate (Berlin et al., 2020). Patients with severe to critical illness display hypoxemia and dyspnea, which may develop into ARDS (Berlin et al., 2020). This stage of the disease is characterized by high circulating levels of pro-inflammatory cytokines, such as interleukin-6 (IL-6), IL-1β, and IL-18 referred to as “cytokine storm” (Mehta et al., 2020), and the development of a prothrombotic state (Iba et al., 2020). Within the group of patients with severe COVID-19, there are significantly more individuals with pre-existing comorbidities of the cardiovascular system like elevated cholesterol levels, high blood pressure or a history of myocardial infarction (Figure 1). Endothelial dysfunction is generally present in infections caused by highly pathogenic Coronaviruses and has been shown to be particularly pronounced in SARS-CoV-2 infections resulting in damage of the pulmonary and other vascular endothelium (Teuwen et al., 2020). This suggests a synergistic activation of vascular inflammatory pathways that are associated with both severe COVID-19 and cardiometabolic diseases (Kadosh et al., 2020). In addition to alveolar damage in COVID-19 patients, vascular wall edema, hyaline thrombi, microhemorrhages, and diffuse thrombosis of peripheral small vessels contribute to disease severity (Carsana et al., 2020; Fox et al., 2020). One reason for vascular cell damage may be the high concentration of circulating proinflammatory cytokines and ferritin in severe COVID-19 patients (Carubbi et al., 2021; Gao et al., 2021). The latter is also reflected by high concentrations of circulating soluble P-selectin in COVID-19 patients who were admitted to intensive care units (ICU) compared to patients who did not require intensive care (Goshua et al., 2020). In addition, elevated levels of thrombomodulin (a membrane-bound regulator of coagulation, which is released during endothelial cell injury) were also associated with an increased risk of mortality in COVID-19 patients (Goshua et al., 2020). Similarly, increase of circulating endothelial cells in patients with COVID-19 correlated with a higher number of platelets and lymphocytes and the inflammatory endothelial marker soluble vascular cell adhesion molecule 1 (sVCAM1) (Guervilly et al., 2020).

**FIGURE 2:**
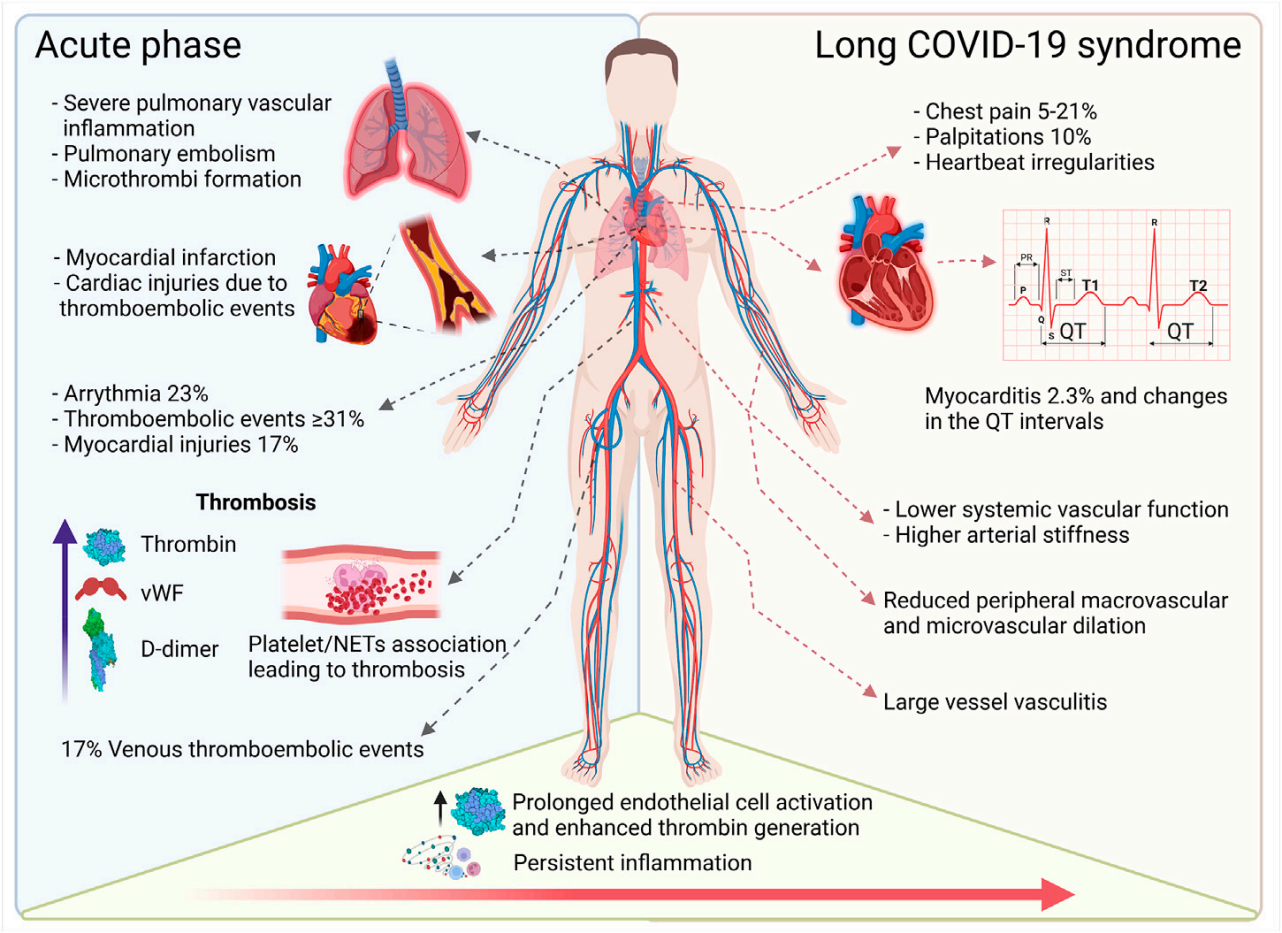
Overview of cardiovascular complications in acute and long-term COVID-19. The acute phase of COVID-19 induces pulmonary, cardiac, and peripheral thrombotic events and can trigger arrythmia and myocardial injury. Persistent inflammation and accompanying vascular dysfunction foster cardiac complications such as palpitations and myocarditis. Ongoing (chronic) vascular inflammation increases vascular stiffness and reduces micro- and macrovascular dilatation (This figure was made with biorender.com).

Taken together, severe COVID-19 may present as a hyperinflammatory prothrombotic disease with multiorgan involvement affecting the entire vasculature. The presence of pre-existing cardiovascular disease (CVD) is associated with an increased risk for a more severe disease course and higher mortality. Moreover, long-term consequences of SARS-CoV-2 infection, now referred to as “Long COVID-19 syndrome (LCS)” (Venkatesan, 2021), negatively impact the heart and the vascular system, however, these effects are poorly understood (Figure 2). Hence, in this review, we will focus on the impact of SARS-CoV-2 infection on the vascular system and detail how this pathogen promotes chronic inflammation and vascular damage, which may contribute to the development of LCS.

**FIGURE 3:**
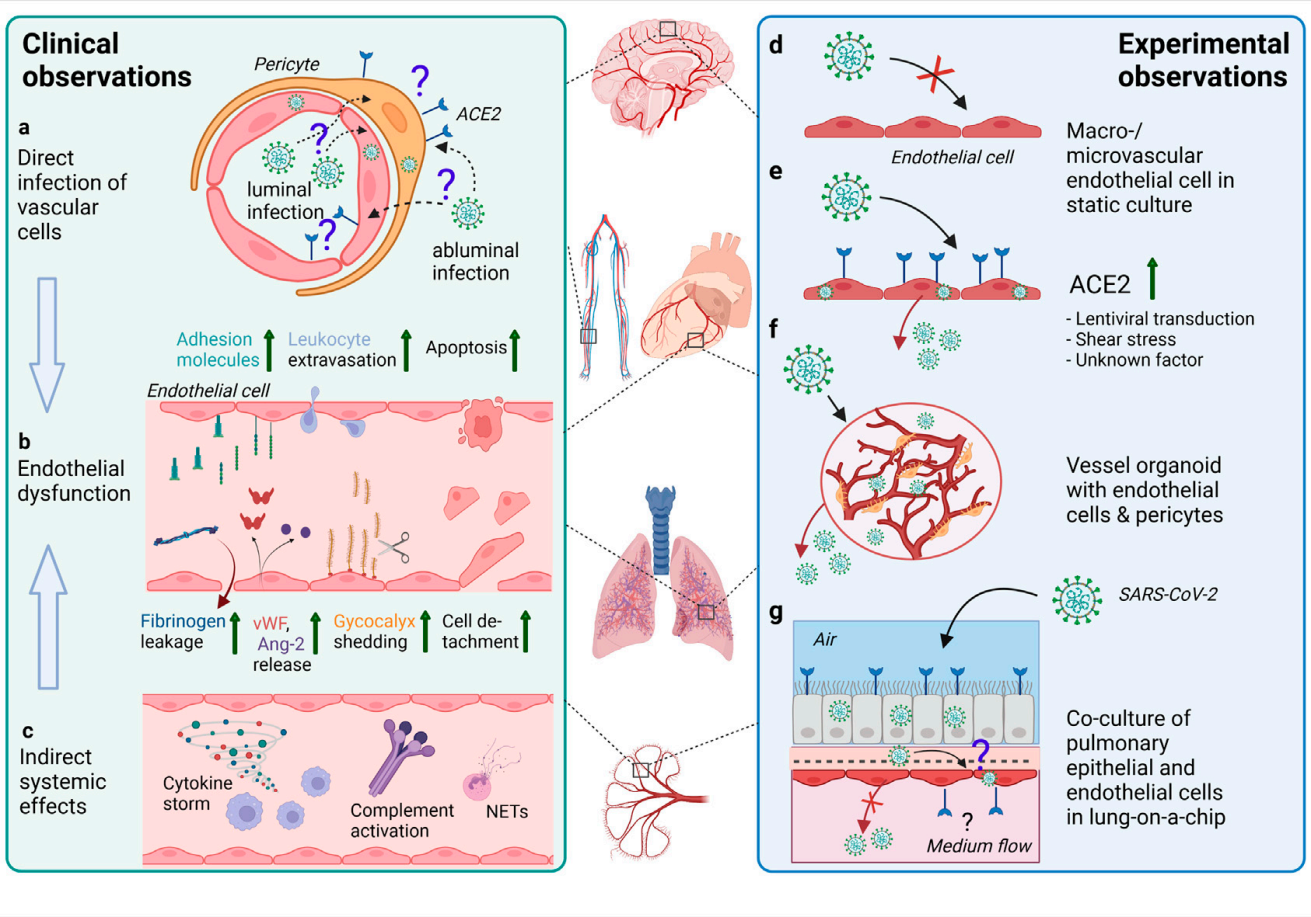
Effects of SARS-CoV-2 infection on the endothelium as observed in patients and different experimental models. **(A)** In endothelial cells in various organs (such as depicted in the middle of the figure) from COVID-19 patients, SARS-CoV-2 RNA or protein has been inconsistently detected. The ACE2 expression is rather found in pericytes than endothelial cells. Infection of vascular cells can happen from the abluminal side at primary sites of infection in the respiratory tract or from the luminal side in case of hematogenous SARS-CoV-2 dissemination to distal organs. To what extent infection of endothelial cells and/or pericytes occurs and contributes to COVID-19 pathology is unclear. **(B)** Endothelial dysfunction is not only observed in the pulmonary vasculature, but throughout the body as a hallmark of severe COVID-19 and may be one of the main contributors to increased frequency of thrombotic events. Elevated levels of markers of endothelial activation and injury including von Willebrand factor (vWF), angiopoietin-2 (Ang-2), soluble forms of several cell adhesion molecules and glycocalyx degradation products, and increased numbers of detached endothelial cells are found in COVID-19 patients’ blood. Postmortem analysis of multiple organs has revealed perivascular immune cell infiltrates, decreased endothelial barrier properties as visualized by fibrinogen leakage and endothelial apoptosis in some of the COVID-19 patients. **(C)** Besides direct infection of vascular cells, immune-mediated mechanisms such as excessive pro-inflammatory cytokine production or complement-hyperactivation can play a major role in causing endothelial dysfunction. **(D)** Macro- and microvascular endothelial cells isolated from different anatomical locations (such as depicted in the middle of the figure) in conventional monolayer culture were not permissive to infection by SARS-CoV-2. **(E)** When ACE2 was introduced into endothelial cells by lentiviral transduction productive infection occurred. ACE2 expression in endothelial cells might also be induced by shear stress or certain interferons. **(F)** In co-culture models such as hiPSC-derived vessel organoids consisting of endothelial cells and pericytes, productive infection occurred, **(G)** whereas in lung-on-a-chip-models infection of the lung epithelial compartment can lead to infection and cytopathogenic effects of the adjacent endothelial cells (This figure was made with biorender.com).

We will first discuss SARS-CoV-2 and its entry route, followed by a summary of what has been described about COVID-19 and CVD. Thereafter, the impact of SARS-CoV-2 on vascular cells will be debated based on what has been reported *in vivo* and *in vitro* (Figure 3). Finally, LCS and its potential impact on cardiovascular health will be summarized.

## 2 SARS-CoV-2

### 2.1 Viral Replication, S (Spike) Protein and Angiotensin-Converting Enzyme 2 (ACE2)

The replication cycle of SARS-CoV-2 is initiated when the S protein binds the entry receptor ACE2 and is subsequently cleaved by a host protease such as TMPRSS2 at the plasma membrane, or endosomal cathepsins, allowing for either direct fusion with the plasma membrane or the endosome (Hoffmann et al., 2020; V’kovski et al., 2021b). Inside the infected cell structural proteins of the virus such as the S and N protein, are generated and, together with the newly formed genomic RNA, assembled (V’kovski et al., 2021b) and released from the infected cell via exocytosis. These newly formed virus particles are ready to infect new cells in the next round of viral infection.

The S protein is a type I transmembrane glycoprotein sharing around 73% sequence identity with SARS-CoV (Lu et al., 2020). Further, it is a trimer composed of subunit 1 (S1), which contains the receptor binding domain (RBD), and the subunit 2, which is linked to S1 through a junction region that is susceptible to cleavage by furin. Cleavage of S2 by TMPRSS2 leads to the exposure of the fusion peptide allowing for fusion of the viral-host membranes facilitating the entrance of the virus into the cell (Hoffmann et al., 2020; Jaimes et al., 2020; Walls et al., 2020; Papa et al., 2021).

Thus, S protein initiates the entrance of the virus to the host cell through direct binding of the RBD with the peptidase domain of ACE2, which is described as the main entry receptor for SARS-CoV-2 (Hoffmann et al., 2020; Yan et al., 2020).

Despite RNA viruses being prone for mutations or adaptations in their viral genome, the evolutionary changes of SARS-CoV-2 have been limited until now (Korber et al., 2020). Most adaptations have been observed in the S protein, with the first report being the single amino acid change D614G (Glycine (G) substitutes Aspartic acid (D) in the amino acid position 614), becoming the first predominant strain circulating in Europe. Compared with the original strain isolated from patients in Wuhan, D614G was able to enhance the spread of the virus including an increased infectivity which resulted in higher viral titers in infected patients and more severe disease outcome (Korber et al., 2020; Onder et al., 2020).

The variants alpha, beta, gamma, delta, and omicron have important mutations in the S protein leading to an increased transmission and/or hospitalization rate, respectively (Li M. et al., 2021; Cameroni et al., 2021; Kumar et al., 2021). Most variations occurred in the RBD resulting in a higher affinity for the receptor ACE2 and in supporting escape from antibody neutralization, this includes for example variants B.1.1.7, B.1.526 and B.1.429 of the alpha lineage (Wang P. et al., 2021; Liu et al., 2021; West et al., 2021). The beta variant B.1.351 was the one responsible for the high transmission rate in South Africa with the highest frequency of mutations located in the S protein, namely 77% (Wang P. et al., 2021). The delta variant B.1.617.2 first described during the second wave of COVID-19 in India, gained particular attention as it demonstrated an increased infectivity allowing a faster virus spreading and enhanced immune evasion due to a reduced susceptibility to antibodies elicited by earlier SARS-CoV-2 variants or the respectively designed vaccines (Augusto et al., 2021; Planas et al., 2021). Omicron (B.1.1.529), now taking over, was first detected in November 2021 and is substantially mutated compared to any previously described SARS-CoV-2 variant, including 37 S protein residue substitutions in the predominant haplotype. This high mutation rate suggests that omicron might escape infection- and vaccine-elicited antibody treatment (Hoffmann M. et al., 2021; Cameroni et al., 2021). Altogether, multiple studies have demonstrated the importance of the S protein in the infectivity and pathogenicity of the SARS-CoV-2. Mutations of the S protein are a key factor determining stability/strength of the S protein and ACE2 interaction thus consequently also spreading and immune evasion.

ACE2, the most important entry receptor of SARS-CoV-2, is a homologue of ACE and is a key anti-inflammatory component of the Renin Aldosterone Angiotensin System (RAAS), an important regulator of blood pressure as well as renal, vascular and myocardial function and physiology (Donoghue et al., 2000; Crackower et al., 2002; Paul et al., 2006; Santos et al., 2018; Beyerstedt et al., 2021; Kuriakose et al., 2021) ACE2 is an anti-inflammatory regulator by converting Ang II into Ang (1–7) and Ang (1–9) (Donoghue et al., 2000; Kuriakose et al., 2021). ACE2 exists in two forms, a transmembrane (tACE2) and a soluble (sACE2) form. The ACE2 ectodomain contains a proteolytic cleavage site for ADAM-17, a disintegrin and metalloprotease that releases sACE2 into the circulation (Kuriakose et al., 2021). sACE2 preserves the N-terminal peptidase domain and is therefore able to interact with the S protein of SARS-CoV-2, resulting in the formation of a complex that can enter the cell through endocytosis via the Angiotensin-II-receptor type 1 (Yeung et al., 2021). This mechanism has also been addressed in a therapeutic manner by increasing the concentration of recombinant sACE2 (rsACE2) in the human circulation to prevent infection. Both sACE2 and rsACE2 scavenge SARS-CoV-2, reducing infectivity in cell culture assays (Cocozza et al., 2020; Glasgow et al., 2020) and in a human vascular organoid model *in vitro* (Monteil et al., 2020). Nevertheless, at the beginning of the COVID-19 pandemic, ACE2 inhibitors or angiotensin receptor blockers, already used for treatment of hypertension, were discussed to have negative effects in COVID-19 patients (Fang et al., 2020; Hammoud et al., 2021), as the treatment leads to an increase in ACE2 expression levels (Hoffmann et al., 2020). Yet, recent clinical data indicate that COVID-19 patients with ongoing hypertension therapy have improved clinical outcomes (Yang G. et al., 2020; Bean et al., 2020; Meng et al., 2020; Safizadeh et al., 2021).

ACE2 is highly expressed together with the TMPRSS2 protease in type II pneumocytes in the lung, in absorptive enterocytes in the intestine, and secretory goblet cells in the nasal epithelium (Lukassen et al., 2020; Ziegler et al., 2020). Both molecules were also shown to be present in heart, cornea, and kidney. These observations might explain why COVID-19 is a systemic disease with multiple tissues involved in the progression and clinical outcome (Hikmet et al., 2020; Sungak et al.,2020).

### 2.2 Virus Detection in Pulmonary and Extrapulmonary Tissue

SARS-CoV-2 is a respiratory pathogen that mainly transmits via aerosols and targets epithelial cells located in the respiratory tract, which represent the main entry port and primary replication site inside the human body (Jonsdottir and Dijkman, 2016). The respiratory tract can be divided into two distinct compartments, namely the upper (from the nasal cavity to the larynx) and lower (from the trachea to the bronchi) respiratory tract, known for their different ambient temperatures of approximately 33 and 37 °C, respectively (Mcfadden et al., 1985). Both compartments are lined with multiple types of epithelial cells including ciliated, mucus-secreting goblet, columnar and basal cells in a pseudostratified manner (Zepp and Morrisey, 2019). The cellular tropism of the SARS-CoV-2 is described to be ciliated (Hou et al., 2020; Ahn et al., 2021; Ravindra et al., 2021; Robinot et al., 2021) or non-ciliated cells (V’kovski et al., 2021a). Infection with SARS-CoV-2 leads to transcriptional alterations in ciliated and non-ciliated cells, which eventually cause activation of the innate immune response and subsequent cytokine release (Chen et al., 2021). In addition, the different temperatures of the upper and lower respiratory tract can have a distinct effect on the replication kinetics. *In vitro*, SARS-CoV-2 infection leads to ±10-fold higher viral titers in well-differentiated airway epithelial cells incubated at 33°C compared to 37°C (V’kovski et al., 2021a). *In vivo*, the higher viral loads might also be linked to an altered innate immune response or to varying cellular host factors that facilitate viral infection (Hoffmann H.-H. et al., 2021; Yan et al., 2021). However, despite its preference for the respiratory tract, SARS-CoV-2 is also observed in other tissues of COVID-19 patients. Similarly, the blood vessels supplying these organs are also prone to damage by SARS-CoV-2-mediated inflammation. Therefore, the following paragraphs will provide a short overview of important organs which may also be targeted by SARS-CoV-2.

*Gastrointestinal Tract*: Viral transcripts could be observed in stool and rectal swabs during later stages of viral infection, suggesting that the oral route is another pathway for viral entry (Parasa et al., 2020; Xiao et al., 2020). Indeed, staining for SARS-CoV-2 N protein in different anatomical regions of the gastrointestinal tract of COVID-19 patients revealed positive signals in the glandular epithelium of the stomach, duodenum, and rectum, but not in the esophagus, which correlated with ACE2 expression of the respective tissue (Xiao et al., 2020).

*Liver*: Immunohistology analysis of liver biopsies taken from COVID-19 patients post-mortem showed that viral RNA can be visualized using *in situ* hybridization in blood clots and in the endothelium of the liver (Sonzogni et al., 2020). Hepatocytes might be susceptible to direct viral infection since syncytium formation and apoptotic cells have been observed in COVID-19 patients, which could also explain the hepatic impairment (Wang Y. et al., 2020).

*Central Nervous System (CNS)*: A broad range of neurological symptoms observed in patients with mild to severe disease such as ageusia, anosmia, headache, fatigue, deficits in cognitive and memory function, delirium or altered mood suggests that SARS-CoV-2 also affects the CNS (Ellul et al., 2020; Pajo et al., 2021; Rogers et al., 2021). Viral RNA and/or protein in various brain regions *post-mortem* has been infrequently and inconsistently detected (Meinhardt et al., 2021; Pajo et al., 2021; Song et al., 2021) and viral RNA has been rarely detected in cerebrospinal fluid of SARS-CoV-2 infected patients (Lewis et al., 2021). Inoculation of human pluripotent stem cell (hPSC)-derived brain organoids with SARS-CoV-2 revealed absent or mostly abortive infection of neurons and/or astrocytes, pointing towards the notion of limited neurotropism and replication in the CNS of SARS-CoV-2 (Ramani et al., 2021). The frequent loss of smell and the high viral load in the olfactory epithelium of COVID-19 patients (Meinhardt et al., 2021) brought the CNS entry route via axons of the olfactory receptor cells into focus, but this has not been confirmed (Khan et al., 2021).

Other potential targets of SARS-COV-2 in the CNS are the choroid plexus epithelial cells (Jacob et al., 2020; Pellegrini et al., 2020; Yang A. C. et al., 2021; Fuchs et al., 2021) brain microvascular endothelial cells (Bhatnagar et al., 2021; Meinhardt et al., 2021; Nuovo et al., 2021; Schwabenland et al., 2021; Song et al., 2021) and pericytes (Bocci et al., 2021).

*Pancreas*: In post-mortem material of COVID-19 patients, the viral N protein was detected in β-cells located in islets of the pancreas, which have been proposed as potential site of viral replication (Müller et al., 2021; Qadir et al., 2021). This resulted in an impaired production of insulin upon stimulation with glucose *in vitro* and could be the reason for the hyperglycemia observed in COVID-19 patients (Zhu L. et al., 2020; Affinati et al., 2021; Montefusco et al., 2021; Müller et al., 2021).

*Kidney*: Acute kidney injury has been observed in COVID-19 patients including collapsing glomerulopathy, heavy proteinuria and podocytopathy (Braun et al., 2020; Cheng et al., 2020; Shetty et al., 2021). Viral transcripts have been detected in all compartments of the kidney with the highest levels found in the glomeruli (Puelles et al., 2020).

To conclude, infection of SARS-CoV-2 is not limited to the respiratory tract. An overview of the different sites of viral infection is provided in the Table 1. Putative direct infections of heart and vasculature will be detailed in Section 3 and Section 4.

**TABLE 1:** Overview of various organs susceptible to viral infection with SARS-CoV-2 and their clinical symptoms in COVID-19 patients.

Organ/tract	Cellular tropism	Viral transcripts detected	Viral immune-staining in material of COVID-19 patients (IF or IHC)	Clinical symptoms in COVID-19 patients	References
Respiratory tract	(Non-) ciliated cells	+	+	Acute respiratory distress syndrome (ARDS)	(Hou et al., 2020
					Ahn et al., 2021;
					Ravindra et al., 2021
					Robinot et al., 2021
					V’kovski et al. 2021a)
Gastrointestinal tract	Glandular epithelium, enterocytes	+	+	Diarrhea, nausea, and vomiting	(Parasa et al., 2020
					Xiao et al., 2020)
Liver	Hepatocytes, Cholangiocytes	+	+	Elevated levels of metabolic enzymes (ALT, AST, GGT), steatosis, fibrosis and cirrhosis, thrombosis	(Lagana et al., 2020
					Sonzogni et al., 2020
					Lopez-Mendez et al., 2021)
Heart	Cardiomyocytes	+	+	Heart injury, arrythmias, myocarditis	(Huang et al., 2020
					Rey et al., 2020
					Coromilas et al., 2021
					Pellegrini et al., 2021)
Pancreas	β-cells	+	+	Pancreatitis, onset type 1 Diabetes Mellitus, ketoacidosis	(Akarsu et al., 2020
					Hadi et al., 2020
					Wang et al., 2020a
					Pandanaboyana et al., 2021)
Kidney	Proximal tubular epithelium, glomeruli	+	+	Glomerulopathy, heavy proteinuria and podocytopathy	(Braun et al., 2020
					Cheng et al., 2020
					Puelles et al., 2020
					Shetty et al., 2021)
Reproductive system	Not determined	?	?	Hypospermatogenesis	(Yang et al., 2020b
					Ma et al., 2021b)
Central nervous system	Not clear	+/−	+/−	Cognitive deficits, headaches	(Meinhardt et al., 2021
					Pajo et al., 2021
					Song et al., 2021)
Vasculature	Endothelial cells; pericytes	—	+	Endothelial dysfunction	(He et al., 2020
					Teuwen et al., 2020
					Varga et al., 2020
					Maccio et al., 2021)

### 3 COVID-19 and Cardiovascular Disease (CVD)

CVD is a class of diseases affecting the heart and blood vessels. CVD includes for example coronary and cerebral artery diseases as well as peripheral artery disease, thromboembolic disease, and venous thrombosis. The most abundant pathophysiology underlying CVD is atherosclerosis, describing a disease of the arterial intima caused by modified lipids and infiltrating inflammatory immune cells mediating endothelial dysfunction. The inflamed intima grows and forms a plaque, which may progress and calcify. Luminal extension of the intimal lesion may lead to vasoconstriction or occlusion while unstable plaques may rupture and cause arterial thrombosis (Soehnlein and Libby, 2021). Causal mechanisms vary but the underlying risk factors for CVD are high blood pressure, smoking, diabetes mellitus, lack of exercise, obesity, and high blood cholesterol - among others. Moreover, age and sex co-determine the individual CVD risk (Stark and Massberg, 2021).

### 3.1 Clinical Observations

CVD manifestations in COVID-19 patients are observed regularly in hospitalized cases. Several types of complications have been identified and their etiology is truly diverse (Chung et al., 2021). Combination of CVD and SARS-CoV-2 infection closely correlates with the severity of COVID-19 progression and mortality (Chung et al., 2021) (Figure 1). These observations were also made with SARS-CoV, which showed similar tendencies (Booth et al., 2003; Chan et al., 2003). Retrospective analysis of COVID-19 patients revealed CVD in 8% to approximately 62% of all hospitalized cases (Zhou P. et al., 2020; Giustino et al., 2020; Sandoval et al., 2020). About one third of all COVID-19 related deaths had underlying CVD (Guo et al., 2020; Onder et al., 2020). The latter suggests a synergistic activation of cardiovascular inflammatory pathways that are associated with both COVID-19 and cardiometabolic disorders (Kadosh et al., 2020). For example, cells of the cardiovascular system, in addition to the respiratory tract, may also represent a direct target for infection with SARS-CoV-2 due to the expression of ACE2 (Groß et al., 2020). ACE2 is also known to be overexpressed in diabetic patients which may facilitate COVID-19 in this population (Cummings et al., 2020; Richardson et al., 2020). Out of 5,700 COVID-19 patients in a cohort from New York, 34% had pre-existing diabetes (Cummings et al., 2020). Further, hypertension and obesity are frequent among Northern Americans (Chobufo et al., 2020), and both comorbidities correlated with a higher mortality of COVID-19 patients (Woolf et al., 2020). Early on in China, the case fatality rate for people with hypertension was about 6 and 10.5% for people with pre-existing CVD (Wu and Mcgoogan, 2020). In a global meta-analysis of 45 studies with a total of 18,300 patients, diabetes was identified as the second common comorbidity after hypertension, and those patients were prompted to a higher risk of in-hospital death independently of age and sex (Silverio et al., 2021). Also Italy reported that patients with comorbidities such as hypertension, hypercholesterolemia, diabetes, and heart disease have an increased risk of mortality (Grasselli et al., 2020; Huang et al., 2020).

### 3.2 Specific Factors of COVID-19 and CVD

*Coagulopathies:* Coagulopathies have also been associated with SARS-CoV-2 infection, independently of pre-existing CVD. In fact, D-dimer levels (a fibrin degradation product used to determine the activation of the coagulation cascade), are enhanced in acute COVID-19 cases (Zhou F. et al., 2020; Berger et al., 2020). Different reports have found a correlation between the increase of D-dimer levels and COVID-19 severity with an about 7-fold increase in critically ill patients, associated with an increased mortality risk (Zhou F. et al., 2020; Yao Y. et al., 2020). In addition, neutrophils and monocytes are strongly contributing to the development of ARDS and thrombosis by inducing hyperinflammation. Neutrophil Extracellular Traps (NETs) are extracellular decondensed chromatin structures mixed with antimicrobial proteins and released in response to infections. Biomarkers of NET formation were increased in the circulation of patients with severe COVID-19 and related to thrombotic events as for example high levels of neutrophil-platelet aggregations were detected (Middleton et al., 2020; Nicolai et al., 2020; Dennison et al., 2021).

Atherosc*lerosis:* Ill-alliance of atherosclerotic endothelial dysfunction and SARS-CoV-2-triggered acute inflammatory responses may accelerate atherosclerotic lesion growth and plaque rupture. This notion is supported by reports from cases of acute myocardial infarction with spontaneous dissection of coronary arteries in patients affected by severe manifestations of COVID-19 (Courand et al., 2020; Long et al., 2020; Romiti et al., 2020). Moreover, statin treatment, which is the gold standard therapy to lower cholesterol levels in CVD patients to limit hyperlipidemia, was shown to reduce in-hospital mortality in patients with diabetes mellitus and COVID-19 (Saeed et al., 2020) and its use was also independently associated with lower intensive care unit admission (Tan et al., 2020). Most likely benefits of statin treatment in this context are rather due to its anti-inflammatory and not to its lipid-lowering function. In addition, it seems that lesion composition does impact on the risk for CVD-associated complications in COVID-19 since higher calcification was correlated with a higher risk of severe COVID-19 (Dillinger et al., 2020). To shed more light on the impact of atherosclerosis for COVID-19 Das and Podder retrieved data of differentially expressed genes for both, atherosclerosis, and COVID-19, from publicly available microarray and RNAseq datasets and performed a protein-protein interaction network analysis. Further functional enrichment revealed inflammatory response genes to be more abundant, particularly MyD88 was identified as a crucial linker of atherosclerosis and COVID-19 (Das and Podder, 2021).

*Non-modifiable risk factors:* Moreover, and similar to CVD, age and sex are risk factors for severe COVID-19 (Fernandez-Martinez et al., 2021; Wehbe et al., 2021). Italy, hit hardly by the first wave in early 2020, reported that patients older than 64 years had an increased mortality risk compared to younger individuals (Grasselli et al., 2020) and people with more than 70 years of age even displayed a case fatality rate of 22.7% (Onder et al., 2020). In addition, available data points towards an increased risk of mortality for male patients with COVID-19 worldwide (Perez-Lopez et al., 2020). An overview of shared comorbidities of CVD and COVID-19 is provided in Figure 1.

### 3.3 COVID-19 and the Heart

According to literature, human cardiomyocytes express only relatively low amounts of ACE2 compared to other heart residing cells such as pericytes or fibroblasts (Chen et al., 2020; Hikmet et al., 2020; Litviňuková et al., 2020; Nicin et al., 2020). Yet, individuals with dilated cardiomyopathy have almost two-fold higher expression of ACE2 in the left ventricle (Bristow et al., 2020). Cardiomyocytes may be directly infected by SARS-CoV-2 or harmed in an indirect manner through the inflammatory response induced by the virus (Yang L. et al., 2021). Human induced pluripotent stem cell-derived cardiomyocytes (hiPSC-CMs) were shown to be susceptible to viral infection (Bojkova et al., 2020; Sharma et al., 2020; Wong et al., 2020; Yang L. et al., 2021; Marchiano et al., 2021) and mimicked cytopathic features of COVID-19 patients’ hearts (Bermejo-Martin et al., 2020; Marchiano et al., 2021). A recent study using an *in vivo* hamster model for SARS-CoV-2 infection showed direct infection of mature cardiomyocytes and revealed that the same mechanism of monocyte recruitment via the chemokine CCL2 occurs *in vivo*, as observed in hiPSC-CMs or in samples from mature cardiomyocytes (Yang L. et al., 2021). Furthermore, active viral replication was reported in infected hiPSC-CMs with a significant increase in the mRNA expression of inflammatory cytokines like IL-6, TNF-α and IL-8 (Wong et al., 2020). Similarly, these cytokines are elevated in COVID-19 patients with co-morbidities such as chronic kidney disease, diabetes, and hypertension (Del Valle et al., 2020). Elevated levels of TNF-α were also measured in patients with congestive heart failure and were shown to contribute to organ damage (Del Valle et al., 2020). SARS-CoV-2 infection also affected the electrical and mechanical function of hiPSC-CMs in the form of a reduced contractile function, lower depolarization of the spike amplitude and lower electrical conduction velocity due to the absence of the Ca2+ flux into the cells (Yang L. et al., 2021; Marchiano et al., 2021).

Initially it was thought that vascular complications in COVID-19 patients, such as small vessel endothelitis and endothelial dysfunction in the heart (Maccio et al., 2021), were caused by direct viral infection of the endothelial cells (discussed in more depth in Section 4). Nevertheless, accumulating evidence now suggests that damage of the vascular system is rather mediated by an augmented inflammatory response (Garnier-Crussard et al., 2020). Indeed, up to 31% of COVID-19 patients in ICU units showed thromboembolic events (Klok et al., 2020), which contributes to the rate of cardiac injuries in the most severely ill COVID-19 patients (Roberts et al., 2020). Other acute cardiac symptoms associated with COVID-19 diagnosis were arrythmias, myocardial injury, and acute heart failure (Rey et al., 2020). Arrythmia was one of the main cardiac symptoms, with 23% of patients who presented with atrial fibrillation (81,8%), followed by bradyarrhythmia (22,6%), and ventricular arrythmia (20,7%) (Rey et al., 2020; Coromilas et al., 2021). Myocardial injuries were reported in 17% of the hospitalized COVID-19 patients and echocardiograms of this group also revealed pericardial effusion and right ventricular myopericarditis (Rauch S. et al., 2020). Echocardiographic analysis elucidated that 13% of patients without a pre-existing CVD condition developed severe cardiac disease, and out of this group 3% progressed to myocardial infarction or myocarditis (Dweck et al., 2020).

Another group of patients prone to get severe COVID-19 are patients with congenital heart disease (CHD) (Lewis et al., 2020; Zareef et al., 2020; Haiduc et al., 2021; Strah et al., 2021). CHD represents one of the most common developmental defects, affecting nearly 1 in 100 newborns and 90% of these patients present with isolated cardiac defects. Notably, although complicated COVID-19 courses are more common in CHD patients, the mortality risk seems not to be significantly affected in adults with CHD and COVID-19 (Strah et al., 2021). In pediatric patients, Down syndrome with common atrioventricular canal is the most described CHD correlating with more severe COVID-19. However, mortality appears to be only increased in patients with aortic stenosis and complex CHD-like hypoplastic left heart syndrome (Strah et al., 2021). In general, larger cohort studies and more in-depth careful evaluations are required to better define subgroups within CHD patients carrying a higher risk of COVID-19 associated mortality.

In conclusion, CVD and COVID-19 form an ill-alliance promoting severe cases (Figure 2). In particular, hypertension and diabetes in the elderly and pre-existing CHD increase the risk for severe disease courses with cardiac involvement. Nevertheless, while hyperinflammation certainly fosters cardiac disease in COVID-19, further studies still need to clarify to which extent mature human cardiomyocyte are susceptible to direct infection with SARS-CoV-2 or whether the observed cardiac effects are mostly due to damage caused by an augmented general inflammatory response.

## 4 COVID-19 and Vascular Cells

### 4.1 General Aspects of Vascular Biology

Endothelial cells line the inner wall of arteries, capillaries, and veins. Although endothelial cells share common properties, they exhibit large phenotypic variability to fulfill their organotypic functions. Their phenotypic heterogeneity arises not only from their position along the vascular tree, but more prominently from their tissue of origin (Aird, 2012; Kalucka et al., 2020; Paik et al., 2020). The endothelium functions as a selective and adaptive barrier to control the exchange of nutrients, metabolites, proteins and cells between the blood and the neighboring tissue. Endothelial cells govern vascular tone and flow through constant production of vasodilating nitric oxide (Humphrey and Schwartz, 2021), and maintain blood fluidity through production of a plethora of anticoagulant and antithrombotic factors (Neubauer and Zieger, 2021). The luminal side of all endothelial cells is covered by the glycocalyx, a non-uniform and complex layer composed of proteoglycans and glycoproteins, that is critically involved in regulation of all endothelial functions (Villalba et al., 2021). On the abluminal side of endothelial cells, perivascular fibroblasts, macrophages, adventitial and mural cells contribute to organotypic functional and structural integrity of vessels (Holm et al., 2018). While large arteries are surrounded by several layers of vascular smooth muscle cells (VSMCs) and perivascular adipose tissue, smaller vessels possess less layers. Venules and veins are incompletely covered with VSMCs, and capillaries and post-capillary venules contain pericytes as the only mural cell type, which are embedded in the same extracellular matrix as the endothelial cells (Van Dijk et al., 2015; Holm et al., 2018). Inadequate control of any of the above-mentioned endothelial cell functions is regarded as endothelial dysfunction and is proposed to be a major contributor to COVID-19 pathology including hypercoagulation (Teuwen et al., 2020; Maruhashi and Higashi, 2021; Nicosia et al., 2021).

### 4.2 Vascular Pathologies During COVID19

#### 4.2.1 Increased Thrombotic Events

COVID-19 is accompanied by a higher incidence rate of venous and arterial thrombotic events compared to historical influenza virus cohorts (Helms et al., 2020; Poissy et al., 2020; Burkhard-Koren et al., 2021). Meta studies comprising several thousands of hospitalized COVID-19 patients estimated average incidence rates of 17% for venous thromboembolic events (VTE) (of which 12% were deep vein thrombosis (DVT) and 7.1% pulmonary embolism (PE)) or 18% for VTE (of which 14% were DVT and 8% PE), respectively (Gratz et al., 2021; Jiménez et al., 2021). In line with the high incidence of thrombotic events, elevated D-dimer levels were found in hospitalized COVID-19 patients (Berger et al., 2020), which often correlated with COVID-19 severity (Tang et al., 2020; Thwaites et al., 2021). D-Dimer levels in COVID-19 patients exceeded the ones found in patients infected with influenza virus (Mei et al., 2020). Another commonly reported parameter that infers the procoagulant state of COVID-19 patients is the imbalance of von Willebrand factor (vWF) and ADAMTS13 (cleaves ultra-large vWF multimers), as evidenced by increased vWF antigen levels and decreased ADAMTS13 activity in hospitalized COVID-19 patients’ plasma (Favaloro et al., 2021). Notably, the increased vWF to ADAMTS13 ratio was accentuated in COVID-19 patients with a fatal outcome (Bazzan et al., 2020; Delrue et al., 2021; Mancini et al., 2021; Rodriguez Rodriguez et al., 2021; Sweeney et al., 2021). Microthrombi, which arise *in situ* in the microvasculature of several organs, have been associated with multiorgan-injury in COVID-19 and are more frequent in patients with arterial hypertension or other comorbidities (Parra-Medina et al., 2021). Several autopsy reports noted the relatively high occurrence of microvascular thrombi in lungs (Ackermann et al., 2020; Carsana et al., 2020; Falasca et al., 2020; Lax et al., 2020; Menter et al., 2020; D’agnillo et al., 2021), heart (Pellegrini et al., 2021; Sang et al., 2021), liver (Rapkiewicz et al., 2020; Kondo et al., 2021), kidney (Rapkiewicz et al., 2020; Akilesh et al., 2021) and the brain (Bryce et al., 2021; Meinhardt et al., 2021; Pajo et al., 2021; Thakur et al., 2021). The finding that VTE, arterial thrombosis and microthrombi co-exist during COVID-19 suggests that these thrombotic events may be driven by several mechanism acting in concert (Gu et al., 2021) including altered platelet function (thrombocytopathy) (Manne et al., 2020; Zaid et al., 2020), endothelial dysfunction (endotheliopathy) (Maruhashi and Higashi, 2021), altered complement function (Stenmark et al., 2021), and features underlying immunothrombosis i.e., increased NET formation (Nicolai et al., 2020; Bonaventura et al., 2021). Here, we focus on the contribution of endothelial cells to COVID-19-associated vasculopathies including thrombotic events.

#### 4.2.2 Clinical Findings of Endothelial Dysfunction in COVID-19 Patients

*Circulating Biomarkers*: Multiple biomarkers indicative of endothelial activation and dysfunction have been found to be elevated in COVID-19 patients with a severe disease course (Lampsas et al., 2021), whereby vascular structural changes have been observed in autopsied organs (Ackermann et al., 2020; Varga et al., 2020; Lee et al., 2021). Pulmonary artery wall thickening due to VSMC hypertrophy, and a decreased lumen size were observed by post-mortem analysis of lungs from COVID-19 patients compared to individuals that were infected with the pandemic A/H1N1/2009 influenza virus (Suzuki et al., 2021). However, most of the studies so far suggest that the endothelium participates in the manifestation and severity of COVID-19. For instance, vWF was significantly increased in the plasma of hospitalized COVID-19 patients indicating endothelial activation and a prothrombotic state in severely ill patients (Fraser et al., 2020; Goshua et al., 2020; Pine et al., 2020; Cotter et al., 2021; Thwaites et al., 2021). Other circulating surrogate markers of endothelial activation such as angiopoetin-2, an autocrine antagonist of Tie-2 receptor promoting vessel-destabilizing effects, was found to correlate with COVID-19 severity (Goshua et al., 2020; Pine et al., 2020; Thwaites et al., 2021) and to be predictive for mortality (Pine et al., 2020). Soluble forms of cell adhesion molecules E- and P-selectin also correlated with COVID-19 severity suggesting type-2 endothelial cell activation in patients with more severe disease (Goshua et al., 2020; Pine et al., 2020; Oliva et al., 2021; Thwaites et al., 2021). Several autopsy studies reported vascular inflammation as seen by perivascular immune cell infiltrates in the lung (Ackermann et al., 2020; Aid et al., 2020; Matschke et al., 2020; D’agnillo et al., 2021; Lee et al., 2021; Maccio et al., 2021; Schwabenland et al., 2021) in some of the COVID-19 patients. Sustained endothelial activation and inflammation may lead to endothelial injury during COVID-19 disease course. Along this line, increased circulating glycocalyx degradation products including syndecan-1, chondroitin sulfate and hyaluronic acid were found in COVID-19 patients and were associated with disease severity (Fraser et al., 2020; Queisser et al., 2021). Moreover, increased activity of glycocalyx modifying enzymes such as heparinase and hyaluronidase were measured (Queisser et al., 2021). In addition, increased numbers of circulating endothelial cells, which putatively detached from the vessel wall due to pathological insults, were found to correlate with COVID-19 severity (Guervilly et al., 2020). Interestingly, elevated circulating endothelial cell frequency persisted in recovered convalescent patients suggesting long-term effects of SARS-CoV-2 infection on vascular function (Chioh et al., 2021). Endothelial cell detachment and apoptosis lead to exposure of pro-thrombotic mediators such as basement membrane proteins,- and abluminally deposited vWF (Neubauer and Zieger, 2021).

*Endothelial Barrier Function*: Altered endothelial barrier properties during SARS-CoV-2 infection were observed by the presence of perivascular immune cell infiltrates. In line with this finding, a damaged alveolar capillary barrier was also visualized by discontinuous immunoreactivity of components of endothelial tight junctions and endothelial basement membrane constituents (D’agnillo et al., 2021). Indicative for a compromised blood-brain barrier, fibrinogen leakage into brain parenchyma was seen in more than 50% of the assessed brains in three case series of COVID-19 patients (Bocci et al., 2021; Lee et al., 2021; Schwabenland et al., 2021). Further, assessment of cerebrospinal fluid from COVID-19 patients presenting with neurological symptoms revealed elevated albumin levels, suggesting that impaired blood-brain barrier or blood-cerebrospinal fluid barrier properties may play a role during the COVID-19 disease course (Bernard-Valnet et al., 2021).

*Complement System*: The complement system is an important part of the innate immune response to bacterial and viral pathogens. Its activation by three different pathways (classical, lectin and alternative) triggers a cascade of enzymatic activation leading to inflammation, phagocytosis and elimination of the pathogen and ultimately results in activation of the adaptive immune response (Merle et al., 2015). Activation of the complement system can also damage endothelial cells, especially if those are already dysfunctional and do not sufficiently express protective, membrane-bound complement regulators (Stenmark et al., 2021). Upregulation of certain complement components in circulation and complement deposition in lung tissue of SARS-CoV-2 infected patients (Cugno et al., 2020; Holter et al., 2020; Magro et al., 2020; Macor et al., 2021) and Rhesus macaques (Aid et al., 2020) were reported. It was shown that SARS-CoV-2 N protein activates the lectin pathway of complement activation (Flude et al., 2021), while others reported that N protein does not activate complement, but S protein activates complement via the alternative pathway (Yu et al., 2020). Interestingly, Ma et al. found that compared to hospitalized patients suffering from influenza virus infection or those with other forms of acute respiratory failure, hospitalized patients with COVID-19 displayed elevated and distinct markers of complement activation (Ma L. et al., 2021). Namely activation via the alternative pathway (as indicated by increased ratio of iC3b:C3, increased factor B, sC5b and Ba concentrations in plasma) and that an increased activation of the alternative pathway also correlated with worse COVID-19 outcome (Ma L. et al., 2021). These studies suggest that complement activation may contribute to endothelial dysfunction and disease severity in COVID-19 (Stenmark et al., 2021).

*Dermal Microvasculature*: Higher incidence rates of cutaneous manifestations such as pernio (chilblain)-like acral lesions compared to pre-pandemic times were associated with mild COVID-19 (Agnihothri and Fox, 2021). Pernio-like acral lesions in COVID-19 patients mostly occur on the toes, are characterized by pink papule that develop into violaceous purpuric lesions and are linked to changes in dermal microvasculature resulting in edema and lymphocyte infiltration (Agnihothri and Fox, 2021). These lesions are not to be confused with skin manifestations observed in severe patients with acral livedoid eruptions and retiform purpura, which arise due to a systemic hypercoagulant state (Agnihothri and Fox, 2021; Do et al., 2021).

Together these studies highlight that vascular cell dysfunction is not only limited to the lung, but the complete COVID-19 pathogenesis shows that the vasculature of multiple other organs are affected as well (Figure 3). However, the specific mechanisms inducing aberrant endothelial function at the different tissue sites only begin to be elucidated. Whether cytopathogenic effects driven by direct endothelial infection with SARS-CoV-2 play a role and which immune-mediated mechanisms are primarily inducing endothelial pathology during COVID-19 is a matter of current investigations. Moreover, whether infection of other vascular cells such as pericytes, VSMC or vascular fibroblasts occurs and contributes to endotheliopathy during COVID-19 is still unclear and needs to be further investigated.

### 4.3 ACE2 Expression in Vascular Cells *in Situ*

The current consensus is that the cell surface expression of ACE2 (and the host protease TMPRSS2) is the main determinant for the cellular tropism of SARS-CoV-2, although additional co-receptors and a number of alternative entry receptors have been proposed (Baggen et al., 2021). Meta-analysis of various recent single-cell RNAseq datasets of mouse brain, heart and lung identified pericytes and certain VSMC populations as the only vascular cell types expressing ACE2, which was corroborated by immunofluorescent analyses, however this data is still preliminary (He et al., 2020). Notably, TMPRSS2 was not detected in the ACE2-positive mural cell types (He et al., 2020). Other investigators also found, by single-cell RNAseq of mouse brain and high-resolution microscopy of cerebral microvessels, that pericytes are the predominant vascular cell type expressing ACE2 (Wenzel et al., 2021). Similarly, in human heart single-cell and single-nucleus RNAseq data sets, pericytes expressed ACE2, while endothelial cells did not (Chen et al., 2020). A meta-analysis of a big number of single-cell and single-nucleus RNAseq data sets from various human tissues revealed that neither endothelial cells nor pericyte and VSMC co-express ACE2 and TMPRSS2 (Muus et al., 2021). Strikingly, endothelial cells showing ACE2 expression also expressed markers of pericytes, suggesting technical difficulties in the separation of endothelial cells from pericytes for transcriptomic analyses and insufficient separation could introduce false-positive ACE2-expression in endothelial cells (Mccracken et al., 2021). While results from RNAseq studies rather point to an absence of ACE2-expression in endothelial cells, several histology-based studies in human post-mortem samples demonstrated scattered expression of ACE2 in presumably endothelial cells. For instance, positive ACE2-immunoreactivity was observed in pulmonary microvascular endothelial cells (Wong et al., 2021) and increasingly with smaller vessel size in the heart (Maccio et al., 2021). ACE2-positive brain microvascular endothelial cells were found in the basal ganglia of COVID-19 and control patients (Kirschenbaum et al., 2021), in the Medulla Oblongata (Meinhardt et al., 2021) and in the frontal cortex predominantly in patients that suffered from dementia or hypertension (Buzhdygan et al., 2020). None of these studies unequivocally showed that vascular ACE2-immunoreactivity indeed stems from endothelial cells, instead the signal could also be pericyte derived. In line, in islet and exocrine capillaries of the human pancreas, ACE2-expression was detected in pericytes (Coate et al., 2020). Another very recent report showed that ACE2 immunolabelling in microvessels of the human frontal cortex coincided with the pericyte marker PDGFR-β and not endothelial CD31 (Bocci et al., 2021). Altogether, expression of ACE2 by endothelial cells is still a matter of debate and whether ACE2 expression in endothelial cells and pericytes differs in various vascular beds, position along the vascular tree or certain (pathological) conditions needs to be investigated in more detail (Figure 3).

### 4.4 Are Endothelial Cells Directly Infected by SARS-CoV-2 *in Vivo*?

In primary sites of infection such as the alveoli, SARS-CoV-2 can reach the lung capillaries at the air-blood barrier from the abluminal side in case of disruption of the alveolar epithelial cell layer. In distant organs endothelial cells can only be targeted by viral infection from the luminal side in case of hematogenous virus spread. Viral RNA in blood (serum or plasma) in COVID-19 patients has been detected most prominently in more severely ill patients (Bermejo-Martin et al., 2020) and blood RNA levels could act as predictor for disease outcome (Li Y. et al., 2021; Hogan et al., 2021; Jacobs et al., 2021; Rodríguez-Serrano et al., 2021). However, it has not been confirmed that viral RNA found in blood also stems from active virions or rather from RNA fragments (Andersson et al., 2020).

Early post-mortem studies suggested that endothelial cell dysfunction in the lung and kidney is directly caused by endothelial cell infection with SARS-CoV-2 (Ackermann et al., 2020; Varga et al., 2020). S protein positivity in CD34 ^+^ cells lining pulmonary vessels, in presumably endothelial cells of the renal glomeruli and the seminiferous tubules was reported (Yao et al., 2021). Co-expression of ACE2 and SARS-CoV-2 spike mRNA was observed by *in situ* hybridization in microvascular cells in the lung of some COVID-19 patients (Wong et al., 2021). Interestingly (D’agnillo et al., 2021), reported infrequent N-protein immunoreactivity in endothelial cells and pericytes of small and medium sized pulmonary vessels exclusively in COVID-19 patients with high viral load and a short time interval between symptom onset and death (D’agnillo et al., 2021). A study employing imaging mass spectrometry of lung sections from COVID-19 deceased patients found S protein positive VSCMs (Rendeiro et al., 2021) indicating that VSMCs could also be a cellular target of SARS-CoV-2. In contrast, other studies reported no detection of SARS-CoV-2 proteins/RNA in pulmonary vessels (Schaefer et al., 2020; Massoth et al., 2021), neither were SARS-CoV-2 proteins detected at any time point in pulmonary vasculature of infected Syrian gold hamsters (Allnoch et al., 2021). In the heart of COVID-19 patients, independent of the occurrence of microthrombi, infected endothelial cells could not be observed, but low numbers of infected myocytes were found (Pellegrini et al., 2021). In contrast, another study that detected clinically relevant SARS-CoV-2 transcripts in 41/95 autopsied hearts, reported the more frequent detection of N protein in ICAM-1^+^ endothelial cells than in alpha-actinin^+^ cardiomyocytes (Brauninger et al., 2021). In brain autopsy case series of COVID-19 deceased patients, detection of viral protein or RNA in brain capillaries in various brain regions such as the brain stem and the olfactory bulb were found in a very low number of patients (Bhatnagar et al., 2021; Meinhardt et al., 2021; Nuovo et al., 2021; Schwabenland et al., 2021; Song et al., 2021).

In summary, viral entry into endothelial cells of (micro)vessels in multiple organs is not a widely observed phenomenon. Since some of the mentioned studies do not use multicolor staining, it is unclear, whether identified viral proteins or RNA are really located in endothelial cells or rather in mural cells, as shown in Figure 3. Another confounding factor is the specificity of antibodies used to detect viral proteins, namely several groups have reported inconclusive results due to positive staining in control tissues (Yang A. C. et al., 2021; Meinhardt et al., 2021; Wong et al., 2021).

### 4.5 *In Vitro* Infection of Endothelial Cells With SARS-CoV-2

#### 4.5.1 Static Cell Cultures

Observations in the vasculature of COVID-19 patients triggered research on endothelial cells *in vitro* to shed light on the possibility of direct viral infection of endothelial cells by SARS-CoV-2. Most of these studies have applied static monolayer cultures of primary human endothelial cells derived from different anatomical locations of the human body using inoculums to initiate the viral infection. For instance, endothelial cell types such as umbilical vein endothelial cells (HUVECs), blood outgrowth and aortic endothelial cells, as well as lung-, brain-, cardiac- and glomerular microvascular endothelial cells, were subjected to viral infection, however, none of these cell were susceptible to direct infection by SARS-CoV-2 (Nascimento Conde et al., 2020; Ahmetaj-Shala et al., 2020). Others showed marginal viral replication in coronary artery endothelial cells after 5 days of infection (Wagner et al., 2021). Moreover, in an established human blood-brain barrier model where CD34 ^+^ umbilical cord blood-derived endothelial cells were grown on filter inserts in co-culture with bovine pericytes, no productive infection of endothelial cells was detected. Also, no impairment of their barrier function was observed, and an inflammatory response remained absent, indicating that these brain-like endothelial cells were not affected by infection with SARS-CoV-2 (Constant et al., 2021). In contrast, although active viral replication remained absent in primary lung endothelial cells, an inflammatory response was induced during SARS-CoV-2 infection, indicating that there is an interaction between primary lung endothelial cells and the virus (Caccuri et al., 2021).

Since direct viral infection of endothelial cells *in vitro* under static conditions is unlikely, as shown in Figure 3, other methods have been applied to make endothelial cells susceptible. One method to infect endothelial cells is to transduce brain- and pulmonary-derived endothelial cells with lentiviruses containing the coding sequence of ACE2, indicating that efficient replication in these cells is possible, but overexpression of ACE2 is required (Nascimento Conde et al., 2020) (Figure 3). Another study has shown with non-physiological inoculums of 10 and 100 multiplicity of infection (MOI) that pulmonary and cardiac endothelial cells show minimal viral replication due to low ACE2 expression (Mccracken et al., 2021). However, infection with these unnatural high amounts of infectious virus particles raises the question if one is still investigating normal entry of the virus or if this might be enforced by alternative pathways like macropinocytosis (de Vries et al., 2011).

#### 4.5.2 More Advanced *in Vitro* and Co-culture Systems

As the infection of static monolayers of endothelial cells was proven to be inefficient, alternative cell culture methods have been applied as well. For instance, transwell filter systems allow for initiation of the viral infection via different compartments, namely apical and basolateral. The study of Schimmel *et al.* (2021) showed that the virus cannot actively replicate in umbilical cord and microvascular endothelial cells, but SARS-CoV-2 is able to enter the cell via either the apical or basolateral side (Schimmel et al., 2021). Moreover, a pro-inflammatory response of the cells was observed, indicative of an interaction of the virus with the cells. In addition, the endothelial cells are exposed to shear stress *in vivo*, and therefore employing microfluidic devices is a potentially interesting technique mimicking the natural vascular environment. Endothelial cells that were seeded in a polydimethylsiloxane (PDMS) channel containing a collagen hydrogel were infected under flow, but also this method did not allow for viral replication of SARS-CoV-2 in the endothelial cells (Schimmel et al., 2021). This is an interesting finding since it has been described that shear stress could upregulate ACE2 expression in brain microvascular endothelial cells, thereby allowing for attachment of the S protein, as shown in (Kaneko et al., 2021) Figure 3.

Another approach is the human lung-on-a-chip model which allows for the culturing of cells in different compartments in an air-liquid interface (ALI, Figure 3). It has been shown for instance that human type I and II alveolar epithelial cells could be cultured in the apical compartment under ALI, while in the basolateral compartment endothelial cells were seeded and exposed to a pulsatile flow (Thacker et al., 2021). Viral infection of the alveolar cells had a direct effect on the endothelial cells by disrupting the confluent cell layer integrity after 3 days of infection, whereby the S protein could be observed in both cell types but viral replication remained absent (Thacker et al., 2021). A different lung-on-a-chip model showed that epithelial cells elicit an inflammatory response after 28 h of infection, while the endothelial chamber did not show a positive signal for the staining of the S protein (Deinhardt-Emmer et al., 2021). Also, the endothelial cell layer remained intact, indicating that these cells were not affected by the infected epithelial cells (Deinhardt-Emmer et al., 2021).

In the vascular wall endothelial cells are accompanied by other cell types like smooth muscle cells and pericytes. Currently, there is minimal knowledge if other vascular cell types play a role in the clinical manifestations in COVID-19-patients, despite their expression of the ACE2 receptor (Hamming et al., 2004; He et al., 2020; Nicin et al., 2020). However, one hypothesis is that the junction leakage of the endothelium is due to the infection of vascular pericytes, which secrete secondary signals that induce activation of microvascular endothelial cells (He et al., 2020). It has been described that iPSC-derived brain pericyte-like cells are susceptible to infection with SARS-CoV-2 whereby viral replication is observed over 72 h (Wang L. et al., 2021). Moreover, iPSC-derived blood vessel organoids containing endothelial cells and pericytes were infected with SARS-CoV-2 and viral replication could be observed on a transcriptional level after 3- and 6-days post infection (Monteil et al., 2020) (Figure 3). Primary human pericytes and astrocytes were directly infected without exhibiting any successful viral replication and cytopathogenic effects (Constant et al., 2021). Altogether, more research should be performed on cells that are present in the vascular wall and perivascular locations since these can have an immense effect on the surrounding tissue, including endothelial cells.

In summary, although the endothelium is severely affected *in vivo* in COVID-19 patients, these observations could not be reenacted *in vitro* in static cell cultures, and more complex cell culture systems in the form of microfluidic channels or lung-on-a-chip models need to be used to detect vascular cell damage or -activation by SARS-CoV-2. In addition, co-cultures of endothelial cells with pericytes showed viral replication, indicating that other cells present in the vascular wall, or the perivascular environment might be susceptible for viral infection and that endothelial cells respond to secondary signals secreted from these infected cells. In this context, the route of infection necessitates consideration. In a situation in which SARS-CoV-2 reaches the vessel via the blood, infection of vascular wall- and perivascular cells would require an endothelial barrier deficiency allowing virus to gain access to these abluminal cell types.

#### 4.5.3 *In Vitro* Investigations of Indirect Mechanisms Affecting Endothelial Function

Recent *in vivo* and *in vitro* findings suggest that direct infection of endothelial cells is rather unlikely. Indirect mechanisms because of epithelial cell infection and exuberated inflammation can play a more important role in causing endothelial damage in COVID-19 (Figure 3). To this end, plasma-induced cytotoxicity in pulmonary vascular endothelial cells 1 h after treatment was assessed and correlated with disease severity and concentration of circulating markers of endothelial damage and organ dysfunction (Rauch A. et al., 2020). Another study did not observe cytotoxicity upon treatment of HUVECs with plasma from hospitalized COVID-19 patients, but observed increased glycocalyx shedding. Concomitantly these COVID-19 plasma samples exhibited elevated heparinase I level (Potje et al., 2021). Treatment of pulmonary microvascular endothelial cells for 16 h with plasma from hospitalized COVID-19 patients induced dysregulated biosynthesis and degradation of endothelial hyaluronic acid. The resulting shift towards lower molecular weight hyaluronic acid fragments in the medium reduced barrier properties of the endothelial cells (Queisser et al., 2021). In contrast, the permeability of brain-like endothelial cells was not changed after 48 h treatment with plasma from COVID-19 patients that developed severe disease (Constant et al., 2021). In severe COVID-19, platelet hyperactivation is often observed (Manne et al., 2020; Zaid et al., 2020), and treatment of endothelial cells with supernatant of activated platelets induced a strong upregulation of pro-inflammatory and procoagulant pathways as assessed by RNAseq. This effect was attributed to the significantly increased transcription of S100A8/A9 in platelets of COVID-19 patients and circulating levels of its protein product MRP8/14, a known pro-inflammatory heterodimer secreted by activated platelets and neutrophils, was also found to correlate with COVID-19 severity (Barrett et al., 2021).

In summary, these studies show that indirect effects of SARS-CoV-2 infection mediated through plasma can be observed also in cultured endothelial cells, highlighting the importance of employing more sophisticated culture system that mimic certain physiological features.

## 5 Long-Covid Syndrome (LCS) in CVD

Around 40–45% of SARS-CoV-2 infections in humans remain symptom free (Oran and Topol, 2020). However, 60–80% of the patients discharged from the hospital have reported at least one residual symptom 50 days after testing positive for SARS-CoV-2 (Carfì et al., 2020; Halpin et al., 2021) and 35% of non-hospitalized individuals reported symptoms 14–21 days after initial diagnosis (Tenforde et al., 2020). Hence, more attention needs to be paid to the potential for long-term complications in patients diagnosed with COVID-19. The term long-Covid Syndrome (LCS) includes a number of different terms such as “Post-acute COVID-19” and “Post-COVID-19 syndrome”. The latter are distinguished according to their duration, into “Post-acute COVID-19″, describing patients who still have symptoms after 4–12 weeks, while patients with symptoms after more than 12 weeks are classified under the “Post-COVID-19 syndrome” (Venkatesan, 2021). The most described symptoms for LCS are fatigue, headache, attention deficit, hair loss, and shortness of breath. In addition, chest pain, palpitations, and tachycardia have also been described, as well as depression and neurologic impairment and dysfunction (Nalbandian et al., 2021). According to current knowledge, these symptoms may persist for months and are also found in patients with mild disease courses (Lopez-Leon et al., 2021; Venkatesan, 2021). With respect to cardiovascular complications studies in deceased COVID-19 patients revealed arterial and venous thromboembolism, strongly suggesting that SARS-CoV-2 negatively affects the vasculature throughout the body with so far largely unknown long-term consequences.

### 5.1 Long Term Consequences of COVID-19 on the Heart

In the context of CVD, chest pain is the most described symptom in patients with prior COVID-19 regardless of severity. Case numbers vary from 5 to 21% two to six months after disease onset with a decreasing trend the longer the disease has been diagnosed (Carvalho-Schneider et al., 2021; Romero-Duarte et al., 2021). Palpitations are also reported with a frequency of about 10% two to six months after COVID-19 diagnosis (Huang et al., 2021; Romero-Duarte et al., 2021) and heartbeat irregularities have raised the question of a more frequent occurrence of arrhythmias after COVID-19. Patients who required ICU treatment had an enhanced average risk of developing acute arrhythmia (Coromilas et al., 2021), but there have been no conclusive studies as to whether these arrhythmias persist in the setting of LCS (longer than 3 months). Palpitations may also indicate tachycardia, but the data on LCS are still limited (Blitshteyn and Whitelaw, 2021), especially since previous studies on e.g., QT interval (time taken for ventricular depolarization and repolarization) changes were also performed in patients receiving drugs that affect these intervals (e.g., chloroquine) *(* O’connell et al., 2021 *)*. COVID-19 patients were also associated with a higher risk of myocardial damage and myocarditis, as magnetic resonance imaging (MRI) screenings revealed pathologic changes in both hospitalized patients and those with mild courses. These changes included increased T1 (spin-lattice relaxation time) and T2 (spin-spin relaxation time) values (associated with heart damage) as well as late gadolinium enhancement (LGE) as retrieved from analysis 30–70 days after COVID-19 diagnosis (Knight et al., 2020; Puntmann et al., 2020). The longest follow-up study was performed 189 days after diagnosis of COVID-19 in 74 patients, but the number of patients with pathologic MRI was very low at that point and T1, T2 and LGE were not significantly changed in this group compared to healthy subjects (Joy et al., 2021). However, the values of affected patients vary widely, which is certainly due to small study cohorts, among other factors (Knight et al., 2020; Puntmann et al., 2020; Kotecha et al., 2021; Pan et al., 2021). Reports with more than 100 patients and analyses for more than 1 month after COVID-19 diagnosis are rare and almost absent for more than 1,000 patients. Therefore, a large study with 1,597 athletes 1 month after COVID-19 diagnosis should be highlighted here. Myocarditis (mostly asymptomatic) was detected in 2.3% of the patients and no significant change in T relaxation time-values was reported (Daniels et al., 2021). Athletes deserve special attention because in this group even a slight decrease in cardiac function, which would be asymptomatic in normal individuals, has a significant impact on performance. Another, population-based analysis, of approximately 35,000 young Americans of both sexes aged 12–19 years revealed a mean myocarditis rate of 0.08%. Around 60% of these cases were diagnosed 19–82 days after infection, however this data is still preliminary (Singer et al., 2021).

In addition, studies have also shown a correlation of elevated serum troponin levels and a higher risk of myocardial injury and substantially higher mortality in COVID-19 patients (Knight et al., 2020; Shi et al., 2020; Wei et al., 2020; Caro-Codón et al., 2021). Notably, in a large study with 1,053 patients with COVID-19 in whom troponin-I, B-type natriuretic peptide, C-reactive protein, ferritin, and D-dimer were measured, only troponin was identified as the only independent predictor of 30-days mortality (Manocha et al., 2021). Nevertheless, earlier studies with a smaller number of patients did report significant changes in D-dimer after an average of 80 days post COVID-19 diagnosis and increases seemed to be more common in hospitalized patients older than 50 years (Townsend et al., 2021; Von Meijenfeldt et al., 2021).

### 5.2 Long Term Consequences of COVID-19 on the Vasculature

Data on long term consequences of COVID-19 disease on vascular function are even more scarce than studies on the heart. Early on during the pandemic researchers learned that acute COVID-19 is associated with severe pulmonary and extrapulmonary vascular inflammation, both on the macro- and microvascular level (Ackermann et al., 2020). In addition, pulmonary and extrapulmonary thromboembolism are common complications, determining initial and maybe also long-term consequences of COVID-19 disease (Madjid et al., 2020). Moreover, some cases of a type 3 hypersensitivity reaction were reported to contribute to vascular inflammation in COVID-19 patients (Roncati et al., 2020) and the highly proinflammatory cytokine response initiated by SARS-CoV-2 is also believed to cause endothelial damage (Cao, 2020). Cytokines like TNF-α and IL-1β are well known for their pro-inflammatory effects on the endothelium and may play a key role in vascular dysfunction in COVID-19 (Norooznezhad and Mansouri, 2021). However, only a few studies with small cohort sizes investigated the long-term impact of COVID-19 on the vasculature. Sollini et al. (2021) evaluated if 2-deoxy-2-[18F]fluoro-D-glucose ([18F]FDG) was able to mark persistent inflammation by examining 10 COVID-19 patients with persisting symptoms for more than 30 days. Imaging was done by [18F]FDG positron emission tomography/computed tomography ([18F]FDG-PET/CT) and showed that the total vascular score was similar in the two groups. However, the target-to-blood pool ratio was significantly higher in three vascular regions (thoracic aorta, right iliac artery, and femoral arteries) in the recovered cohort compared to the control group, arguing for a persisting vascular inflammation. The authors further suggest that the distinct feature (smooth linear pattern) of [18F]FDG vascular uptake in LCS was similar to that observed in large vessel vasculitis (Sollini et al., 2021). Analysis of acute phase markers, endothelial cell activation, NET formation, and thrombin generation in 50 patients 68 days after confirmed SARS-CoV-2 infection showed sustained endothelial cell activation up to 10 weeks following acute SARS-CoV-2 infection (Fogarty et al., 2021). These data further suggest that endothelial dysfunction occurs independently of ongoing acute phase response or NET formation but is associated with enhanced thrombin generation. The authors further hypothesize that shedding of thrombin from endothelial cells may play a role in modulating the loss of normal endothelial cell quiescence (Fogarty et al., 2021). Others investigated the potential effects of SARS-CoV-2 on the systemic vasculature in the arms and legs, examining 20 young adults. Using a cross-sectional design, these two studies assessed vascular function 3–4 weeks after SARS-CoV-2 infection by Doppler ultrasound measuring flow mediated dilation (FMD) in the arm and single passive limb movement in the leg. In addition, carotid-femoral pulse wave velocity was assessed as a marker of arterial stiffness. Results demonstrated significantly lower systemic vascular function and higher arterial stiffness in participants testing positive for SARS-CoV-2 compared with controls. These studies included male and female participants but did not see sex specific effects (Ratchford et al., 2021; Szeghy et al., 2021). In a similar study design but with a longer follow up (3 months from diagnosis of COVID-19) 16 young adults were investigated for brachial FMD, cerebral vasodilator function and arterial stiffness. Out of these 16 participants, eight were still symptomatic while the others did no longer display signs of COVID-19. Subsequent analysis revealed that peripheral macrovascular and microvascular vasodilation was significantly reduced in young adults still being symptomatic, while asymptomatic participants had similar vascular function compared with controls. Cerebral vascular function and central arterial stiffness were unaffected irrespective of COVID-19 symptoms persisting or not (Nandadeva et al., 2021).

In summary, because of the high rate of clinically significant cardiovascular events during acute COVID-19, long-term adverse events are expected (Nalbandian et al., 2021) (Figure 2). However, these need to be better understood, studied in larger cohorts, and most importantly, observed over a longer period of time after infection to better distinguish between effects that resolve after 3–12 months or truly result in chronic disease.

## 6 Summary and Conclusion

Taken together, clinical manifestations of SARS-CoV-2 infection have been detected in several vascular beds (e.g., those of the lungs, heart, and kidneys) and are not restricted to the pulmonary system. According to current research, direct viral infection of cardiomyocytes and pericytes together with dysfunctional endothelium foster vascular dysfunction in COVID-19 patients. Endothelial cells, putatively not prone to direct viral infection through SARS-CoV-2, probably get indirectly activated via the inflammatory immune response (“cytokine storm”) mediated by the virus. In addition, pre-existing CVD renders COVID-19 patients particularly vulnerable for downstream vascular complications and COVID-19-associated mortality. Altogether, chronic inflammation and vascular damage contribute to the acute pathophysiology of COVID-19 but may also cause development of LCS.

Currently, significant efforts are being made to decipher both the direct and indirect impact of SARS-CoV-2 on the vasculature. *In vitro* studies, for example of endothelial cells, pericytes and cardiomyocytes have already shed some light on how SARS-CoV-2 directly affects vascular cells and explored which downstream signaling events are involved. Yet, only limited conclusions on the contribution of immune cells to vascular dysfunction during SARS-CoV-2 infection can be drawn. More complex cell culture/organoid systems to better exploit and resemble the complex nature of cell-cell interactions need to be established *in vitro*. To complement these *in vitro* studies and allow for exploration of long-term consequences of dysfunctional vasculature in COVID-19 well-defined *in vivo* models need to be investigated. Hence, combined efforts are required to comprehend the varied responses to SARS-CoV-2 infection to pave the way for new treatment strategies, including those for the long-term cardiovascular effects of COVID-19.

## Abbreviations

+ , positive; − , negative; IF, Immunofluorescence; IHC, Immunohistochemistry; ALT, Alanine transaminase; AST, Aspartate transaminase; GGT, Gamma-glutamyl transferase.
